# Identification of a Major QTL and Candidate Gene Analysis of Salt Tolerance at the Bud Burst Stage in Rice (*Oryza sativa* L.) Using QTL-Seq and RNA-Seq

**DOI:** 10.1186/s12284-020-00416-1

**Published:** 2020-08-10

**Authors:** Lei Lei, Hongliang Zheng, Yanli Bi, Luomiao Yang, Hualong Liu, Jingguo Wang, Jian Sun, Hongwei Zhao, Xianwei Li, Jiaming Li, Yongcai Lai, Detang Zou

**Affiliations:** 1 Key Laboratory of Germplasm Enhancement, Physiology and Ecology of Food Crops in Cold Region, Ministry of Education, Northeast Agricultural University, Harbin, 150030 China; 2 Heilongjiang Academy of Agricultural Sciences Postdoctoral Programme, Harbin, 150030 China

**Keywords:** *Oryza sativa* L, Salt tolerance, QTL-seq, RNA-seq, Candidate gene

## Abstract

**Background:**

Salt stress is one of the main abiotic stresses that limits rice production worldwide. Rice salt tolerance at the bud burst stage directly affects the seedling survival rate and the final yield in the direct seeding cultivation model. However, the reports on quantitative trait locus (QTL) mapping and map-based cloning for salt tolerance at the bud burst stage are limited.

**Results:**

Here, an F_2:3_ population derived from a cross between IR36 (salt-sensitive) and Weiguo (salt-tolerant) was used to identify salt-tolerant QTL interval at the bud burst stage using a whole-genome sequencing-based QTL-seq containing 40 extreme salt-tolerant and 40 extreme salt-sensitive individuals. A major QTL, *qRSL7*, related to relative shoot length (RSL) was detected on chromosome 7 using ΔSNP index algorithms and Euclidean Distance (ED) algorithms. According to single nucleotide polymorphisms (SNPs) between the parents, 25 Kompetitive allele-specific PCR (KASP) markers were developed near *qRSL7*, and regional QTL mapping was performed using 199 individuals from the F_2:3_ population. We then confirmed and narrowed down *qRSL7* to a 222 kb genome interval. Additionally, RNA sequencing (RNA-seq) was performed for IR36 and Weiguo at 36 h after salt stress and control condition at the bud burst stage, and 5 differentially expressed genes (DEGs) were detected in the candidate region. The qRT-PCR results showed the same expression patterns as the RNA-seq data. Furthermore, sequence analysis revealed a 1 bp Indel difference in *Os07g0569700* (*OsSAP16*) between IR36 and Weiguo. *OsSAP16* encodes a stress-associated protein whose expression is increased under drought stress.

**Conclusion:**

These results indicate that *OsSAP16* was the candidate gene of *qRSL7*. The results is useful for gene cloning of *qRSL7* and for improving the salt tolerance of rice varieties by marker assisted selection (MAS).

## Background

Salt stress is the main factor that restricts rice production because it reduces crop yield and limits agricultural land utilization (Hossain et al. 2015). Moreover, salinized soil has been increasing due to industrial pollution and unreasonable irrigation (Qadir et al. 2014). In recent years, the direct seeding method has become an important cultivation model due to the advantages of low labor intensity and high efficiency. Therefore, salt tolerance at the bud burst stage is a major factor that determines growth stability in salinized soil when adopting the direct seeding cultivation model in rice (Dingkuhn et al. 1992). Sensitivity of rice to salt stress at the bud burst stage leads to a decrease in seedling rate, resulting in lower yields (Zeng et al. 2001). Thus, studying the genetic mechanism of rice salt tolerance at the bud burst stage, and utilizing salt tolerance-related genes for breeding rice varieties suitable for direct seeding in salinized soil is of great importance.

Rice salt tolerance is a quantitative trait controlled by multiple genes, with a complex genetic mechanism (Zheng et al. 2015). To date, with the development of molecular marker technology and the construction of high-density linkage map, more than 900 rice salt-tolerant quantitative trait loci (QTLs) and 140 genes in response to salt stress have been identified on 12 chromosomes, mainly concentrated at the seedling stage and the field growth stage (http://gramene.org/). For instance, *SKC1* (Ren et al. 2005), *Saltol* (Thomson et al. 2010), and *DST* (Huang et al. 2009) are the major genes associated with seedling stage under salt stress. There are few studies on salt-tolerance QTLs at the seed germination stage. In one case, an F_2:9_ recombinant inbred lines (RILs) population was used to analyze the imbibition rate and germination percentage of rice seeds under salt stress and control condition, and a total of 16 QTLs were detected, 4 of which explained more than 30% of the phenotypic variation (Wang et al. 2011). However, the reports on QTLs/gene mining for salt tolerance at the bud burst stage in rice are limited, and as rice seeds are often used after the germination stage by direct seeding in salinized soil, exploring the major QTL/gene of rice salt tolerance at the bud burst stage is of great significance to provide a theoretical and material foundation for their map-based cloning.

Compared to traditional QTL mapping, bulked segregant analysis (BSA) can effectively identify the molecular markers linked to genes or QTLs by generating two DNA bulks with significantly separated phenotypic traits (Michelmore et al. 1991). With the release of sequenced genomes, QTL-seq (Takagi et al. 2013) uses a combination of BSA and next-generation sequencing technologies to accelerate the identification of candidate genes without using markers or obtaining genotyping. To date, many loci have been identified by QTL-seq in different plants including arabidopsis (Schneeberger et al. 2009), soybean (Song et al. 2017), wheat (Trick et al. 2012), sorghum (Han et al. 2015), tomato (Liu et al. 2019) and cucumber (Lu et al. 2014). In rice, several QTLs associated with salt tolerance have also been identified by QTL-seq, such as mutant lines from which an extremely salt-tolerant pool was selected after the heading stage, leading to the identification of the salt-tolerant gene, *OsRR22* (Takagi et al. 2015). Two bulks were created using extreme phenotypes of rice yield traits at the reproductive stage under salt stress using the RIL population developed from CSR11/MI48 and 5021 polymorphic loci and 34 QTL regions were revealed by 50 k SNP chip, in which 7 new salt tolerance QTLs were identified (Tiwari et al. 2016). These reports suggest that it is feasible to identify salt tolerant QTL/genes at the bud burst stage using QTL-seq.

Transcriptome analysis (RNA-seq) has high resolution and sensitivity, and can characterize the responses of various plant species to environmental stresses (Wang et al. 2009). Furthermore, RNA-seq can be used to find new transcripts and rare transcripts, and the data shows high reproducibility with respect to both technical and biological replications (Hoen et al. 2013). With the development of high-throughput sequencing technology, RNA-seq has been widely used to investigate the molecular mechanism of salt tolerance in rice (Shankar et al. 2016; Zhou et al. 2016), cotton (Guo et al. 2015), Leymus chinensis (Sun et al. 2013), and other crops (Hu et al. 2015; Liang and Schnable 2016). However, thousands of differentially expressed genes (DEGs) are identified by RNA-seq, suggesting that it is still difficult to identify the target genes. Recently, a method of combining QTL-seq and RNA-seq was developed, and it can identify chromosome fragments or genes that can be verified by each other. Lately, a major QTL on chromosome 6 for capsaicinoid content was identified using QTL-seq and high-density genetic maps, and candidate genes were revealed by combining QTL-analysis and RNA-seq (Park et al. 2019). The gene, *qPH7*, was identified in a 300 kb interval by QTL-seq in maize, and *Zm00001d020874* was chosen as the target gene by RNA-seq (Zhang et al. 2019). *AcPMS1*, a candidate gene associated with DNA mismatch repair for the restorer-of-fertility in onion was identified using BSA-seq and RNA-seq (Kim et al. 2015).

In this study, QTL-seq was employed to identify the QTLs for salt tolerance at the bud burst stage of rice by an F_2:3_ population derived from a cross between IR36 (salt-sensitive) and Weiguo (salt-tolerant). A major QTL, *qRSL7*, controlling relative shoot length (RSL) at the bud burst stage under salt stress was detected on chromosome 7. Regional linkage mapping analysis narrowed the QTL region down to a 222 kb interval, which contained 5 candidate genes that showed significantly different expression levels between the two parents as revealed by RNA-seq. The qRT-PCR and sequence analysis showed *OsSAP16* was the candidate gene controlling the RSL in rice. This result will be useful for improving the salt tolerance and developing direct seeding of rice in salinized soil.

## Results

### Screening and Evaluation of the Index for Two Mixed Pools

**Fig. 1:**
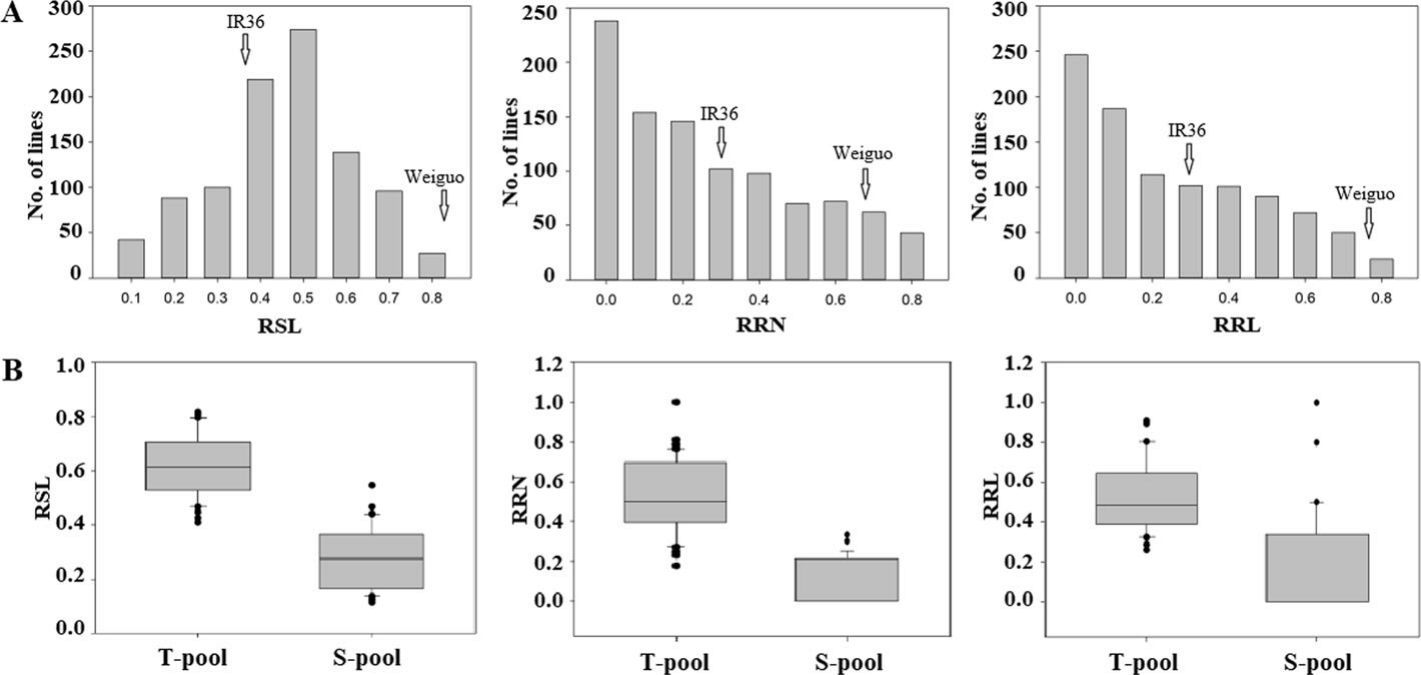
**a** Phenotypic distribution of RSL, RRN and RRL of 983 F_2:3_ lines. **b** Box-plot of phenotypic statistical of RSL, RRN and RRL in two pools. RSL: Relative shoot length; RRN: Relative root number; RRL: Relative root length. Arrows indicate the phenotypic value of IR36 and Weiguo

**Fig. 2:**
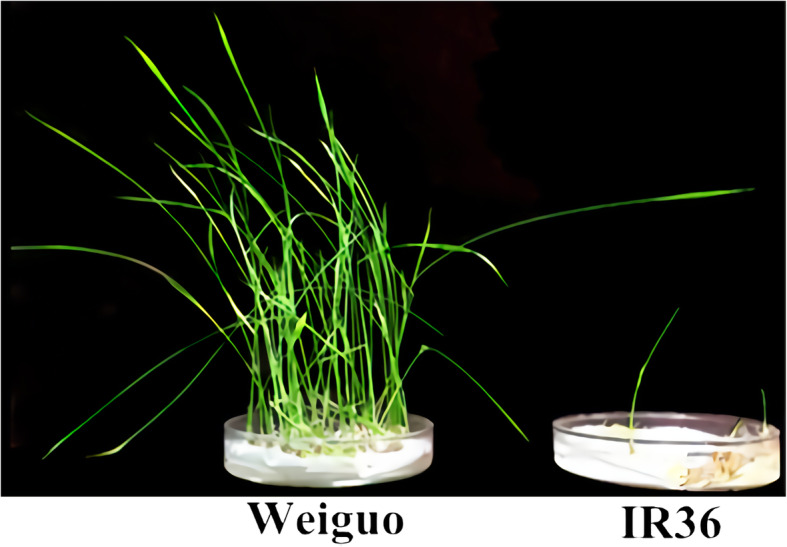
The phenotype of Weiguo and IR36 after 10 days of 0.5% NaCl treatment

**Fig. 3:**
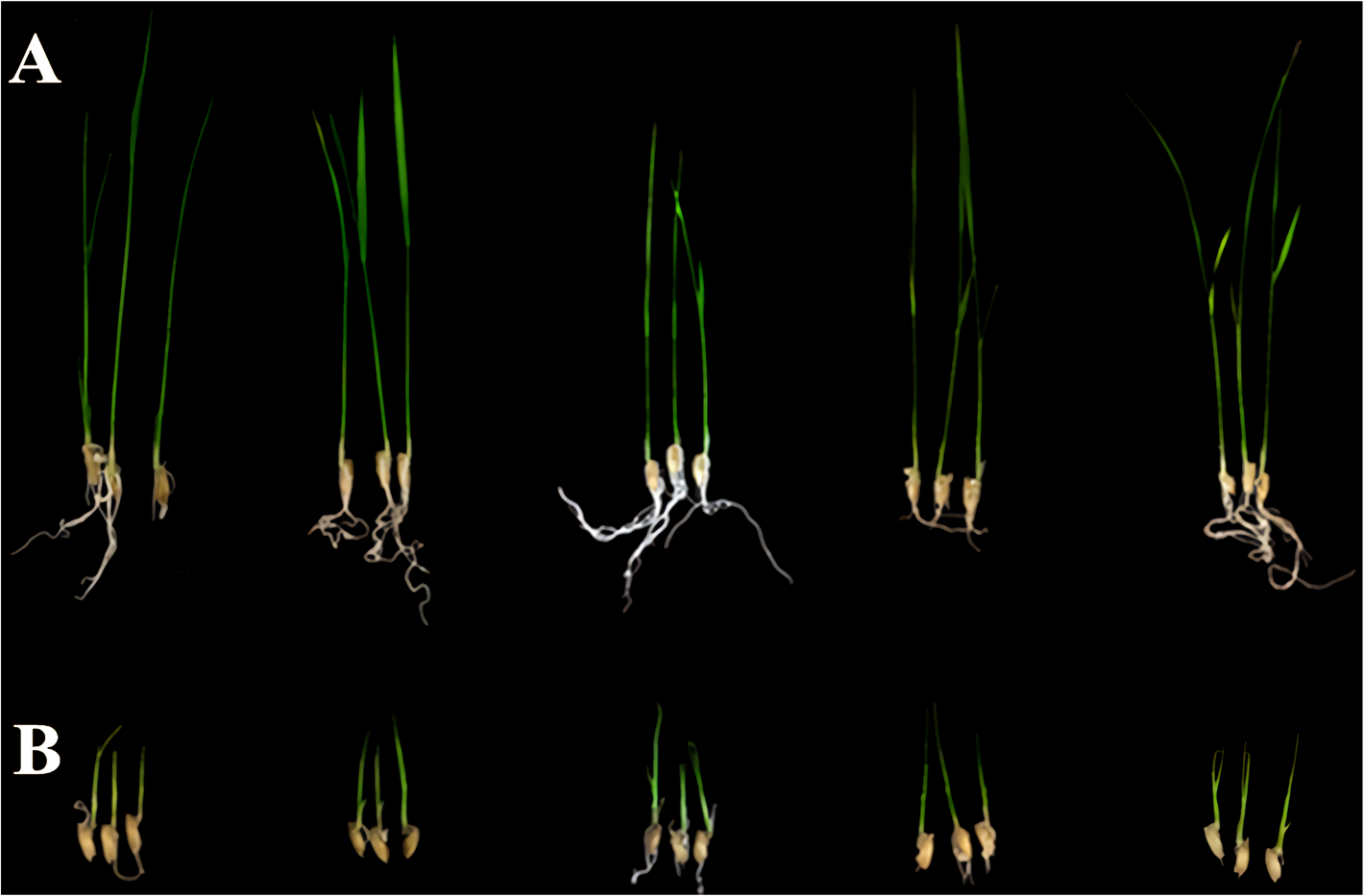
**a** The phenotype of 5 F_2:3_ plants randomly selected from T-pool after 10 days of 0.5% NaCl treatment. **b** The phenotype of 5 F_2:3_ plants randomly selected from S-pool after 10 days of 0.5% NaCl treatment

In the present study, IR36 (salt-sensitive) and Weiguo (salt-tolerant) were crossed to develop an F_2:3_ population containing 983 lines for QTL-seq analysis. The shoot length (SL), root number (RN), root length (RL), and their relative values in 983 individuals were evaluated under salt stress and control condition at the bud burst stage. Only the relative shoot length (RSL) showed a normal distribution, whereas the relative root number (RRN) and relative root length (RRL) exhibited skewed distribution (Fig. 1a), which was not in accordance with the inheritance of quantitative traits. More than 200 individuals showed the value of both RRN and RRL at 0 (Fig. 1a), which was due to slow or stopped root growth in most lines after salt stress. Thus, using RSL as the screening trait for the two mixed pools, 40 lines with extreme salt tolerance and 40 lines with extreme salt sensitivity were selected to prepare the tolerant pool (T-pool) and sensitive pool (S-pool), respectively, which were then used for DNA sequencing. The RSL were significantly lower in the S-pool than in the T-pool, indicating that the overall phenotypic data of the S-pool was greatly affected by salt stress (Fig. 1b). Moreover, the key to plant survival under NaCl salt stress is to maintain a low Na^+^/K^+^ ratio in cells (Wang et al. 2012). To verify the accuracy of the selected extreme lines for T-pool and S-pool, we determined the Na^+^/K^+^ ratio of shoots (SNK), and found that the SNK was lower in the T-pool than in the S-pool (Additional file 1: Figure S1). In addition, the phenotypic statistics of RRN and RRL also proved the exactitude of the extreme lines selection for the two pools (Fig. 1b). The phenotype of the two parents after salt treatment is displayed in Fig. 2, while Fig. 3 shows the phenotype of the 5 F_2:3_ plants randomly selected from the T-pool and S-pool after salt treatment.

### Sequencing of the Parents and Extreme Pools

A total of 141 Gbp clean reads were obtained from the two parents and the two F_2_ pools, and the Q30 all reached 85%. The average sequencing depth was 27.60X, and the average mapped ratio and the average genome coverage was 98.95% and 96.21%, respectively (Additional file 2: Table S1). GATK software was used to analyze the SNPs in the results. According to the SNPs obtained from the comparison of the parents and the two F_2_ pools with Nipponbare reference genome, a total of 4,825,004 SNPs were detected between IR36 and Weiguo, including 6395 non-coding transcript variants (Additional file 2: Table S2). In addition, 1,085,342 SNPs were detected between the T-pool and S-pool, and these contained 1740 non-coding transcript SNPs (Additional file 2: Table S2). An association analysis was performed using the SNPs detected between the T-pool and S-pool.

### QTL-Seq Analysis Combining SNP-Index Algorithms and ED Algorithms

**Fig. 4:**
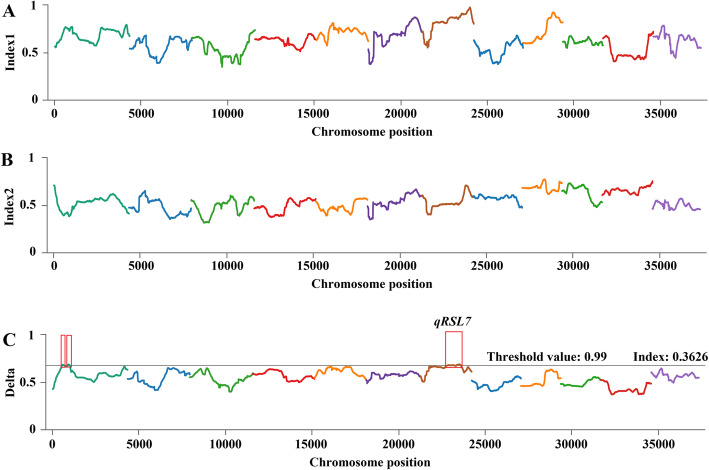
ΔSNP-index algorithm mapping salt-tolerant QTL. X-axis represents the position of 12 chromosomes of rice and Y-axis represents the SNP-index or ΔSNP-index. The different colour lines show the SNP-index or ΔSNP-index value of fitting results in 12 chromosomes. **a** The SNP-index graph of T-pool. **b** The SNP-index graph of S-pool. **c** The ΔSNP-index graph

**Fig. 5:**
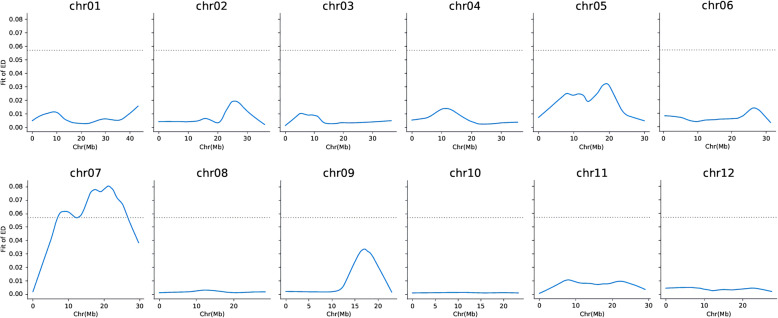
ED algorithm mapping salt-tolerant QTL. The blue line represents the fitting value of ED, and the dashed line represents the threshold line

We used two algorithms to detect the QTL associated with salt tolerance at the bud burst stage. As shown in Fig. 4 and Table 1, a total of three regions were detected by the ΔSNP-index method, which were located on chromosome 1, 1, and 7, and the size of the regions was 2.68 Mb, 2.92 Mb, and 4.17 Mb respectively. A total of two regions were detected by the ED method, and both were located on chromosome 7, and the physical distances were 5.17 Mb and 14.13 Mb, respectively (Fig. 5, Table 1). By taking overlapping regions into account, these two methods yielded a region of 20,160,000–24,330,000 bp on chromosome 7, named *qRSL7*, which was considered as the candidate region related to salt tolerance at the bud stage in rice (Table 1). Through analysis of the gene sequence in this interval, 16,415 SNPs and 2719 Indels were found, of which the number of SNPs and Indels causing amino acid changes was 554 and 50, respectively. Within the 4.17 Mb region, 532 genes were predicted based on the RAP-DB database (http://rapdb.dna.affrc.go.jp/) (Additional file 2: Table S3).

**Table 1:** Distribution of salt-tolerant region on chromosomes detected by ΔSNP-index and ED algorithms

Method	Chromosome	Start (bp)	End (bp)	Size (Mb)
ΔSNP-Index	1	4,590,000	7,270,000	2.68
	1	7,800,000	10,720,000	2.92
	7	20,160,000	24,330,000	4.17
ED	7	6,942,377	12,109,211	5.17
	7	12,547,138	26,674,659	14.13

### Regional Linkage Mapping Analysis

**Fig. 6:**
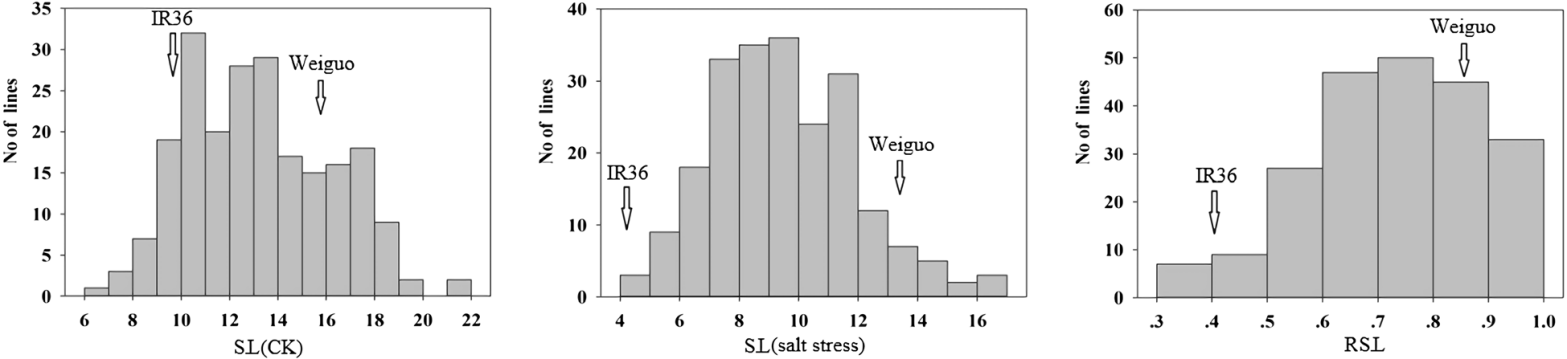
Phenotypic distribution of SL under salt stress and CK and RSL of 199 F_2:3_ lines. SL: Shoot length; RSL: Relative Shoot length; CK represents the lines growth in water at the bud burst stage. Arrows indicate the phenotypic value of IR36 and Weiguo

As *qRSL7* still contained a large number of genes, we developed 25 KASP markers near this region and used them for genotyping 199 F_2:3_ lines randomly selected from 983 individuals of the F_2:3_ population. Under the condition of salt stress and control, the SL and its relative values of the parents and the 199 F_2:3_ lines were counted (Additional file 2: Table S4). The phenotype values of SL under salt stress and control condition and RSL were all significantly different between the parents (Additional file 2: Table S4), and the 199 F_2:3_ lines showed continuous distribution under control and salt stress (Fig. 6). The parents’ phenotype values were between the extreme values of the 199 F_2:3_ individuals, which showed transgressive segregation (Fig. 6). The absolute values of skewness and kurtosis were both less than 1, indicating that the data of RSL were suitable for QTL analysis (Additional file 2: Table S4).

**Fig. 7:**
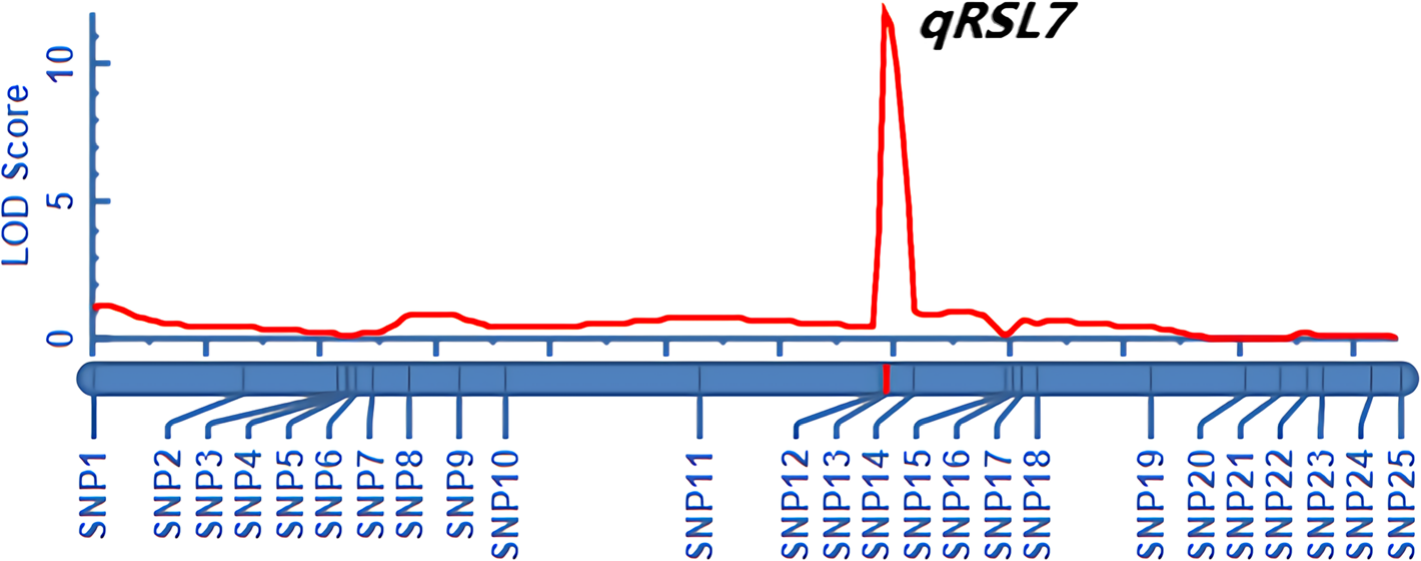
*qRSL7* detected in the F_2:3_ population contained 199 lines by QTL ICIMapping4.2 software

After calculation using ICIMapping4.2 software, a linkage interval of 222 kb was obtained on chromosome 7 (Fig. 7). As shown in Table 2, the RSL was linked to the SNP13-SNP14 marker interval. *qRSL7* explained 24.90% of the phenotypic variation, and the LOD value and additive effect was 11.84 and − 0.12, respectively. The positive allele of the QTL was contributed by Weiguo (Table 2). Thus, the interval of *qRSL7* was optimized from 4.17 Mb to 222 kb by constructing a genetic map and 27 genes were contained in this region (Additional file 2: Table S3).

**Table 2:** Detection information of *qRSL7* detected by regional linkage mapping

Trait name	Chromosome	QTL	Left marker	Right marker	LOD	PVE(%)	Add
RSL	7	*qRSL7*	SNP13	SNP14	11.84	24.90	−0.12

### RNA-Seq Statistics

To identify DEGs between the parents, we performed transcriptome analysis of IR36 and Weiguo at 36 h after salt stress and control condition at the bud burst stage. In total, 23.77 million, 23.95 million, 23.70 million, and 24.17 million clean data were obtained from the IR36 and Weiguo transcriptome libraries under the normal condition (control) (marked as IR and WG) and salt treatment (marked as TIR and TWG), respectively. The high-quality base (% ≥ Q30) of each sample were all more than 88.88% (Additional file 2: Table S5). The alignment results showed that 75.82–82.38% of the clean reads from all twelve samples could be mapped to the reference genome. On average, approximately 34.45 (72.57%) and 37.49 (77.92%) million reads were uniquely mapped to the reference genome using TopHat for IR36 and Weiguo, respectively (Additional file 2: Table S6).

### Classification of DEGs

Four comparative groups (TWG vs. TIR, WG vs. IR, IR vs. TIR, and WG vs. TWG) were constructed by comparing the same rice cultivar under different conditions (control and stress) and the samples of different rice cultivars (IR36 and Weiguo) under the same condition. Additional file 3: Figure S2 shows the volcano plots of DEGs in the four groups. By restricting -log10 (FDR) > 1.3 (FDR < 0.05), there were a total of 3227, 4177, 5921, and 1519 significant DEGs in TWG vs. TIR, WG vs. IR, IR vs. TIR, and WG vs. TWG, respectively. The results revealed that there were significant differences in the gene expression level within the varieties as well as the treatments. Within the two comparison groups of different varieties, 1150 DEGs were up-regulated and 2077 DEGs were down-regulated among the 3227 DEGs in TWG vs. TIR, and there were 1220 and 2957 DEGs that were up-regulated and down-regulated, respectively, among the 4177 DEGs in WG vs. IR. Furthermore, 3984 DEGs were up-regulated and 1937 were down-regulated in IR vs. TIR while 1237 were up-regulated and 282 were down-regulated in WG vs. TWG (Additional file 3: Figure S2). Obviously, more DEGs were up-regulated in IR vs. TIR. In addition, more DEGs in the salt-sensitive variety responded to salt stress compared to those in the salt-tolerant variety under salt conditions (Additional file 3: Figure S2). This result showed that the effect of salt stress on the salt-sensitive variety was greater than that on the salt-tolerant variety. In total, 9708 unique DEGs were detected in all four groups. These DEGs could be divided into 15 disjointed subgroups, among them 6.19% (601/9708), 10.88% (1056/9708), 34.06% (3307/9708), and 2.79% (271/9708) were group-specific DEGs in TWG vs. TIR, WG vs. IR, IR vs. TIR, and WG vs. TWG, respectively. Additional file 4: Figure S3 shows the Venn diagram of the 9708 unique DEGs in these four groups.

### Gene Ontology (GO) and Kyoto Encyclopedia of Genes and Genomes (KEGG) Pathway Enrichment Analysis

For all DEGs in the four comparison groups, a total of 2383 (73.85%), 3119 (74.67%), 4842 (81.78%), and 1317 (86.7%) DEGs were assigned GO terms in TWG vs. TIR, WG vs. IR, IR vs. TIR, and WG vs. TWG, respectively. Obviously, DEGs identified in IR vs. TIR were assigned significantly higher GO terms than those in WG vs. TWG. The cellular protein metabolic process was the most significant in the biological process category, indicating that the rice bud under salt treatment had wide metabolic activities. In the cellular component category and molecular function category, the cell part and catalytic activity were the most significantly represented group, respectively (Additional file 5: Figure S4).

To further study the DEGs involved and enriched in various metabolic pathways, the KEGG pathway database was used for pathway-based analysis. The results revealed that 485 of 3227 DEGs in the TWG vs. TIR, 782 of 4177 DEGs in the WG vs. IR, 1407 of 5921 DEGs in the IR vs. TIR, and 401 of 1519 DEGs in the WG vs. TWG were annotated, respectively (Additional file 6: Figure S5). Among the significantly enriched pathways, the phenylpropanoid biosynthesis pathway contained more DEGs than the others. This result suggested that the phenylpropanoid biosynthesis pathway might have a modulating effect on salt-responsive gene expression. These annotations will provide valuable information for research on the salt stress response pathways in rice (Additional file 6: Figure S5).

### Candidate Gene Analysis

QTL-seq analysis revealed a common QTL named *qRSL7* (Table 1), and the interval was reduced from 4.17 Mb to 222 kb by regional linkage mapping analysis (Fig. 7). Within the 222 kb region, 27 genes were predicted based on the RAP-DB database (http://rapdb.dna.affrc.go.jp/) (Additional file 2: Table S3). To detect the candidate genes, we combined the QTL-seq and RNA-seq results, and only selected the DEGs in IR vs. TIR and WG vs. TWG within the candidate region. Among the 27 predicted genes, 5 genes were differentially expressed under salt stress (Additional file 2: Table S7). Therefore, these 5 DEGs were selected as candidate genes for further confirmation. In addition, three genes, namely *Os07g0569166*, *Os07g0569700*, and *Os07g0572075*, were also differentially expressed in WG vs. IR or TWG vs. TIR, and *Os07g0570575* was significantly down-regulated in both WG vs. IR and TWG vs. TIR. In contrast, *Os07g0570500* showed no significant up- or down-regulation in WG vs. IR or TWG vs. TIR (Additional file 2: Table S7).

**Fig. 8:**
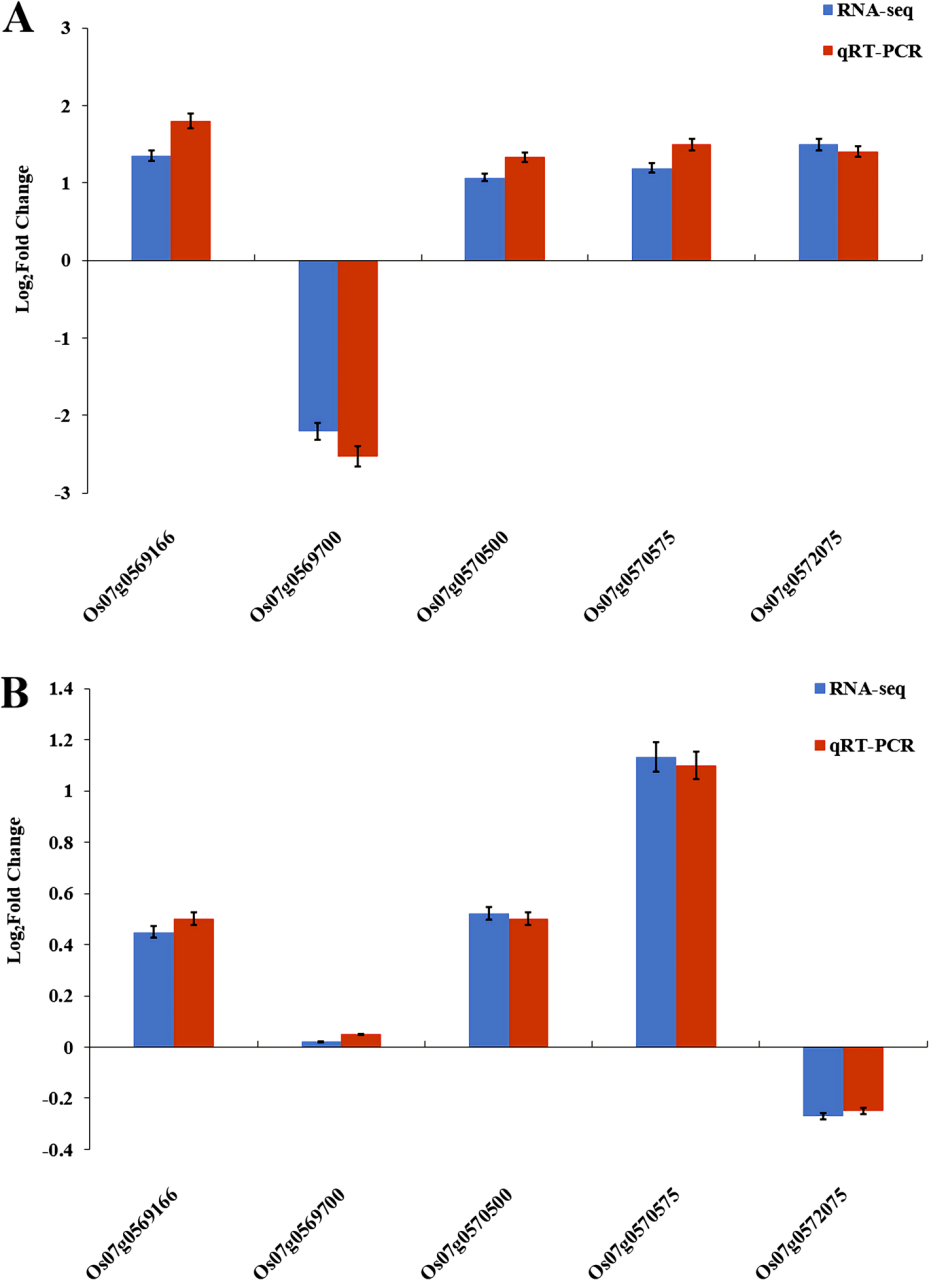
Comparison of RNA-seq results and qRT-PCR analysis of gene expression levels. **a** Log_2_ fold change of candidate genes in IR vs. TIR. **b** Log_2_ fold change of candidate genes in WG vs. TWG. Blue and red block represent RNA-seq results and qRT-PCR analysis, respectively

To confirm the accuracy and reproducibility of Illumina RNA-Seq results, the 5 genes were compared for their expression levels between IR36 and Weiguo by quantitative real-time PCR (qRT-PCR) analysis under normal and salt stress conditions. The validation results for the 5 genes are shown in Fig. 8. Based on the RNA-seq results, the four genes *Os07g0569166*, *Os07g0570500*, *Os07g0570575*, and *Os07g0572075* were all up-regulated in expression in TIR compared with IR. Moreover, *Os07g0569700* was down-regulated in IR vs. TIR. There were no genes that were significantly differentially expressed in WG vs. TWG. The relative trends in the expression patterns of the qRT-PCR results were all consistent with the RNA-seq data, but the absolute expression levels showed some differences (Fig. 8). Furthermore, to validate the expression of the 5 genes in the F_2:3_ population, we respectively selected 5 individuals with high, low and intermediate phenotype of RSL from the 199 F_2:3_ population. Under the salt stress treatment, *Os07g0569700* was down-regulated and the other 4 genes were up-regulated in low RSL lines, and no significant up- or down-regulation was observed in high and intermediate RSL lines (Fold change: expression data of salt treatment/expression data of control) (Additional file 7: Figure S6).

### Identification of Candidate Genes Responsible for Salt Tolerance of Rice

**Fig. 9:**
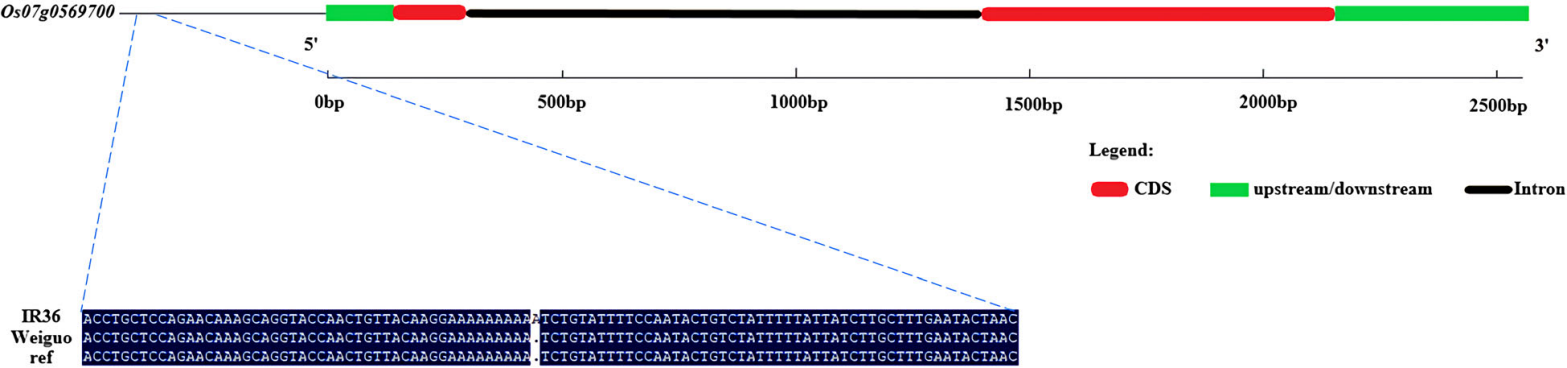
Sequence difference analysis of *Os07g0569700*. The gene structure of *Os07g0569700* and sequence differences in *Os07g0569700* between IR36 and Weiguo. Ref is the reference sequence of Nipponbare genome

To further obtain strong evidence to determine the most possible candidate gene, the 5 candidate genes were sequenced in IR36 and Weiguo. Sequence analysis exhibited no difference in the cDNA sequence and promoter region of *Os07g0569166*, *Os07g0570575*, and *Os07g0572075* between IR36 and Weiguo. *Os07g0570500* showed that in the CDS region, one SNP (T to C) was detected in IR36 compared with Weiguo (ATG start codon 96 bp downstream); however, we found that the SNP caused no amino acid changes using ProtParam (https://web.expasy.org/protparam/). Only *Os07g0569700* showed that in the promoter region, IR36 contained a 1 bp insertion (ATG start codon 879 bp upstream) compared with Weiguo (Fig. 9). Next, we designed a KASP marker using the 1 bp insertion sequence to genotype the two parents and 199 F_2:3_ lines, which were also used for regional linkage mapping analysis. The KASP genotyping result is shown in Additional file 2: Table S8. The correlation analysis of female parent genotype, male parent genotype, and heterozygous genotype with RSL suggested that there was a significant positive correlation between the genotype and phenotype (Additional file 8: Figure S7). And, the results of ANOVA revealed that the differences between female parent genotype and male parent genotype, female parent genotype and heterozygous genotype, and male parent genotype and heterozygous genotype were all significant (F > F-crit, *P* value < 0.01; Additional file 2: Table S9). These suggested that the KASP maker was significantly associated with salt tolerance at the bud burst stage in the F_2:3_ population under salt stress.

Therefore, *Os07g0569700* was considered as the most possible functional gene underlying *qRSL7*. The candidate gene *Os07g0569700* has been studied and named as *OsSAP16* in a prior study (Wang et al. 2016). This gene encodes a stress-associated protein and its expression has been shown to increase under drought stress.

## Discussion

Soil salt stress is one of the main abiotic stresses that affects crop yields, and seed germination, root length, and plant height is significantly inhibited under salt treatment (Liang et al. 2014). If rice plants are sensitive to salt stress at the bud burst stage, the seedling rate will be decreased, which will reduce the rice yield. However, the studies on QTLs/gene mining of rice after germination by direct seeding in salinized soil are limited. The salt tolerance of 295 rice germplasm resources was evaluated and extreme salt tolerance variety (Weiguo) /salt sensitive variety (IR36) were selected by our laboratory. Furthermore, IR36, an *indica* rice, is a recognized salt-sensitive variety that has been widely applied to the study of salt tolerance of rice (Khan et al. 2016; Yao et al. 2005). In the present study, an F_2:3_ population derived from a cross between IR36 and Weiguo, a salt-tolerant *japonica* rice cultivar, was evaluated with respect to the SL, RN, and RL, and their relative values at the bud burst stage under salt stress and control (water) condition to collect individuals of two pools. The salt stress was induced using 0.5% NaCl based on a preliminary experiment with 3 replicates, and the phenotypes of the lines treated with 0.5% NaCl were found to be significantly different. All the measures used in this research could effectively obtain accurate SL, RN, and RL phenotypic data.

The traditional QTL fine mapping and map-based cloning are limited by high-density genetic map and a series of near-isogenic lines. For example, based on conventional mapping methods, 8 years were required for a predecessor to isolate the major salt-tolerant gene, *qSE3* (He et al. 2019; Cheng et al. 2016). QTL-seq uses a combination of BSA and next-generation sequencing technologies to rapidly identify the chromosome region harboring the genes/QTLs of interest. For example, the salt-tolerant gene *OsRR22* was isolated in an F_2_ population from a cross between the salt-tolerant mutant *hst1* and wild-type Hitomebore using BSA-Seq analysis, which only required 2 years (Takagi et al. 2015). In this study, we identified a major QTL (*qRSL7*) using QTL-seq and rapidly optimized it by regional linkage mapping analysis from 4.17 Mb to 222 kb to explain 24.90% of the phenotypic variation. Therefore, our research provides an effective strategy for the rapid identification of major salt-tolerant QTL at the bud burst stage. Compared with previous researches, some loci for salt tolerance have been reported near/harboring *qRSL7* in recent years. *qPH7.1 s*, a 4.34 Mb interval linked with plant height at the reproductive stage under salt stress (Mohammadi et al. 2013), was overlapped with *qRSL7* in this study. *qGP7–2* (Wang et al. 2011) and *qSTS7* (Tian et al. 2011), two QTLs, identified to control the germination percentage and salt tolerance score, respectively, were approximately 1.0 kb and 29.3 kb distant to *qRSL7*; however, their phenotypic variation explanation (PVE) was only 9.2% and 10%, respectively, which was much lower than that of *qRSL7* (24.90%). These results indicate that of all the QTLs related to salt tolerance found on rice chromosome 7, *qRSL7* is a major QTL related to salt tolerance at the bud burst stage in rice. Beyond these, we did not find any QTL loci close to our interval on chromosome 7.

Transcriptome analysis can be used to characterize various plant species responses to environmental stresses (Wang et al. 2009). In our study, 5 DEGs within the *qRSL7* interval were found using RNA-seq (Additional file 2: Table S7). Among them, *OsSAP16* showed a 1 bp difference in the promoter region between IR36 and Weiguo and was significantly associated with the salt-tolerant phenotype. This gene encoded a C2H2-type zinc finger protein and would be induced under drought stress (Wang et al. 2016)*.* Furthermore, according to the transcriptome analysis and qRT-PCR results, the candidate gene *OsSAP16* was significantly down-regulated after salt stress in IR vs. TIR (Fig. 8), revealing that the expression of this gene was inhibited by salt treatment in IR36.

To date, there have been no reports regarding *OsSAP16* in rice salt tolerance. However, upon searching the Arabidopsis Information Resource (TAIR, http://www.arabidopsis.org/) database using the gene sequence of *OsSAP16*, two Arabidopsis homologous genes were identified (*AT2G41835* and *AT3G57480*) (Additional file 2: Table S10), and only *AT3G57480* was annotated with the function of “response to salt” in TAIR, suggesting that it may be involved in regulating the reactions to salt in Arabidopsis. Thus, we have a conclusion that *OsSAP16* is the candidate gene for *qRSL7*.

In this study, IR36 and Weiguo are *indica* and *japonica* rice varieties, respectively. To prove that the 1 bp Indel is specific for salt tolerance between *indica* and *japonica* rice varieties, we investigated the genotype of this 1 bp Indel in 66 diverse rice accessions based on the data of pan-genome analysis (Zhao et al. 2018). The results showed that 16 of the 66 rice accessions contained this 1 bp insertion, and among these, 15 rice accessions were *O. sativa indica* (including GP51, GP777–1, HP119, HP263, HP274, HP327, HP517–1, HP362–2, HP383, HP396, HP407, HP486, HP492, HP577, and GLA4) and the remaining rice accession was *O. sativa aus*, which was a subgroup of *indica* rice (Uraguchi et al. 2014); one remaining *indica* accession named W0128 was not detected any results (Additional file 2: Table S11). These results indicate that this 1 bp insertion is probably specific to *O. sativa indica* and is associated with the decreased salt tolerance in IR36 at the bud burst stage. These data indicate that *OsSAP16* is the strong candidate gene of *qRSL7*. However, genetic transformation, and further studies are needed to confirm this conclusion.

## Conclusion

In this study, QTL-seq, regional linkage mapping analysis, and transcriptome analysis were performed to identify the genes for salt tolerance at the bud burst stage in an F_2:3_ population derived from a cross between a salt-sensitive variety, IR36, and a salt-tolerant variety, Weiguo. A major QTL, *qRSL7*, associated with salt tolerance at the bud burst stage in rice was located at the 222 kb interval on chromosome 7, which contained 5 candidate genes that showed significantly different expression between the two parents. Based on the qRT-PCR and sequence analysis, *OsSAP16* was found to be the candidate gene controlling the RSL in rice. This study provides a fast and cost-effective strategy to identify salt-tolerant genes at the bud burst stage in rice.

## Methods

### Plant Materials

The two extreme rice cultivars, IR36 (salt-sensitive) as the female parent and Weiguo (salt-tolerant) as the male parent were crossed to harvest the F_1_ population, and an F_2_ population was subsequently generated by selfing the F_1_ population. All plants were grown at the experimental station of Northeast Agricultural University. The leaves of 983 F_2_ lines collected at the active tillering stage, were stored at − 80 °C for DNA extraction, and the corresponding F_2:3_ seeds were collected for further experiments.

### Selection of Salt-Sensitive/Salt-Tolerant Individuals at the Bud Burst Stage

Two DNA bulks for sequencing were constructed by selecting extreme individuals from the F_2:3_ mapping population of 983 lines. Before this, 20 individuals were randomly selected from 983 lines to evaluate their salt tolerance together with the parents in 0.25%, 0.5%, 0.75%, 1% NaCl and water (control), respectively. The phenotypes of the lines treated with 0.5% NaCl were significantly different. Therefore, we selected 0.5% NaCl to evaluate salt tolerance. The individuals were dried at 40 °C for 2 days to break the seed dormancy. Then, 120 full seeds which were randomly selected from each material, were divided into control and salt treatment on an average, and placed evenly in petri dishes. The seeds were then surface-sterilized with 2% NaClO_3_ for 10 min and rinsed thrice with sterile water. Next, the seeds were soaked in water at 30 °C for germination, and when most seed buds were equal to half the length of the seed, 50 uniformly germinated seeds were selected in each petri dish and treated with 0.5% NaCl or an equivalent amount of distilled water. The experiment was repeated three times. The temperature of the climatic cabinate was adjusted to 28 °C/12 h (day) and 25 °C/12 h (night) with relative humidity set at 60%. After 10 days of 0.5% NaCl treatment, 5 plants of each material were randomly selected to measure the SL, RN, and RL under the control and salt treatment condition. Then RSL, RRN, and RRL were further calculated to evaluate salt tolerance at the bud burst stage. Relative value = (phenotype value under treatment condition) / (phenotype value under control condition). Moreover, the shoots of the selected extreme lines for T-pool and S-pool were harvested. The samples were dried at 80 °C for 2 days and then 0.1 g of each dried sample was ground and digested with 0.1 N Nitric acid (Fisher Scientific) at 70 °C for 8 h (Campbell et al. 2017). The concentrations of Na^+^ and K^+^ of shoots were analyzed by a flame photometer (Sherwood410, Cambridge, UK) and then SNK was further calculated.

### QTL-Seq Analysis

Genomic DNA was extracted from the leaves of parent plants, IR36 and Weiguo, and from extreme individuals (40 extreme salt tolerance and 40 extreme salt sensitivity) in two pools using the cetyltrimeth-ylammonium bromide (CTAB) method with minor modifications (Pahlich and Gerlitz 1980). DNA quality was determined using the NanoPhotometer® spectrophotometer (IMPLEN, CA, USA) with a required concentration greater than 50 ng/μl. Next, the DNA was ultrasonically fragmented into 500 bp using a Covaris S2 (Covaris) and was used for constructing DNA libraries with an NEBNext DNA Library Prep Reagent Set for Illumina (BioLabs). The DNA libraries were then sequenced on an Illumina HiSeq X Ten platform at the Beijing Genomics Institute (BGI). Clean reads from both parents and the two DNA pools were compared with the Nipponbare reference genome (Takuji 2005) using BWA software (Li and Durbin 2009). Reads of the T-pool and S-pool were separately aligned to Weiguo and IR36 consensus sequence reads to call SNPs with the SAM tools software (Li and Durbin 2009). PCR duplicates were removed using MarkDuplands tool in Picard, and GATK software was utilized for quality control and filtering to ensure the accuracy of SNPs. The SNP-index association algorithm was used for calculating the genotype frequency differences between two pools. A SNP index represented the proportion of reads containing the SNP different from the reference sequence. The ΔSNP-index of each locus was calculated by subtracting the SNP-index of the T-pool from that of the S-pool (Takagi et al. 2013). The ED algorithm was used to search markers with significant differences between the pools based on sequencing data and evaluation of relevant regions between the markers and traits. The equation of ED algorithm was as follows:

$$ ED=\sqrt{{\left({A}_{aa}-{A}_{ab}\right)}^2+{\left({C}_{aa}-{C}_{ab}\right)}^2+{\left({G}_{aa}-{G}_{ab}\right)}^2+{\left({T}_{aa}-{T}_{ab}\right)}^2} $$

where A_aa_, C_aa_, G_aa_ and T_aa_ separately correspond to the frequency of bases A, C, G and T in the T-pool. A_ab_, C_ab_,G_ab_ and T_ab_ separately correspond to the frequency of bases A, C, G and T in the S-pool. The depth of each base in two pools and the ED value of each SNP loci were calculated. To eliminate the background noise, the fit of ED was used as the associated value (Hill et al. 2013). The correlation interval was obtained by the ΔSNP-index algorithm and ED algorithm, and the common region associated with the two algorithms was the considered as the candidate region.

### Regional Linkage Mapping Analysis

In total, 199 individuals were randomly selected from the F_2:3_ mapping population to evaluate salt tolerance by RSL, and the identification methods used were the same as those for the selection of salt-sensitive/salt-tolerant individuals. According to the SNPs near the QTL region obtained from QTL-seq, Primer 5.0 was used to design the 25 KASP markers (Additional file 2: Table S12) to genotype 201 individuals, including 199 from the F_2_ population and two parents. KASP assays were conducted in a 1536-well plate format using the protocol of LGC Genomics (LGC, Middlesex, UK) with the following PCR protocol: 94 °C for 15 min, 95 °C for 20 s, 55 °C for 60 s (− 1 °C/cycle, 10 cycles in total) and 94 °C for 10 s; 57 °C for 60 s (30 cycles). The KASP reaction mixture system is shown in Additional file 2: Table S13 and the KASP reaction was set as described previously (Liu et al. 2019) with minor modifications. The Synergy H1 full-function microplate reader (FLUO star Omega, BMG Labtech, Germany) was used to read the fluorescence signal. Then, the linkage group was constructed by ICIMapping 4.2, and the grouping module LOD > 3.0 was set to detect the linkage of the markers. Genetic distance was calculated using Kosambi function and the linkage map was drawn with MapChart2.2 (Voorrips 2002). QTL analysis was conducted by inclusive composite interval mapping (ICIM) in ICIMapping 4.2 (http://www.isbreeding.net); LOD ≥ 3.0 was selected as the threshold to determine QTL existence, and the significance level was set at 0.05 (Churchill and Doerge 1994).

### RNA Extraction and RNA-Seq

The two parents, IR36 and Weiguo, were treated with 0.5% NaCl at the bud burst stage, and the control was set at the same time. The buds of the treatment group and the control group (3 repeats) treated for 36 h were sampled and immediately frozen in liquid nitrogen. Total RNA was isolated from the buds using a TranZol Up RNA kit (TransGen Biotech). RNA purity was determined using the NanoPhotometer® spectrophotometer (IMPLEN, CA, USA) and the A260/A280 ratios of all of samples were between 1.8 and 2.0. RNA integrity was detected using an Agilent 2100 Bioanalyzer, and no sign of degradation was found. cDNA was prepared from total RNA using the HiFiScript cDNA Synthesis Kit (CoWin Biosciences, Beijing, China). The cDNA library construction and Illumina sequencing were carried out following the manufacturer’s recommendations (Illumina, San Diego, CA, USA) and the library quality was evaluated on an Agilent Bioanalyzer 2100 system. In total, 12 samples were sequenced. Clean reads were obtained by removing the adaptor sequences and low-quality sequences from the raw data, and mapping to the reference genome using TopHat v2.0.12 with the default parameters. The reference genome and gene model annotation files were downloaded from the Rice Genome Annotation Project (http://rice.plantbiology. msu.edu). All downstream analyses were based on clean data with high-quality reads. Differences in gene expression between the two samples were tested by the cuffdiff program. GO annotation of candidate genes was carried out using agriGO (Tian et al. 2017). Kyoto Encyclopedia of Genes and Genomes (KEGG) pathway enrichment analyses were performed using the KEGG database (http://www.genome.jp/kegg/).

### Quantitative Real-Time PCR Analysis and Candidate Gene Prediction

Quantitative real-time PCR (qRT-PCR) was conducted to confirm the accuracy and reproducibility of the Illumina RNA-Seq results. qRT-PCR gene accessions and primer sequences are provided in Additional file 2: Table S14. Using 2 × Fast qPCR Master Mixture (DINING, Beijing, China) on the Analytik Jena qTOWER system (German) to performed qRT-PCR. Three biological replicates, each with three technical replicates were examined per sample. *Actin-EFα1* was used as the internal control. Relative gene expression levels were calculated using the 2^-ΔΔCt^ method (Livak and Schmittgen 2000). The methods for RNA extraction and qRT-PCR of the 5 respective individuals with high, low, and intermediate phenotype of RSL from the 199 F_2:3_ population were the same as above.

According to QTL-seq and linkage mapping analysis verification, a 222 kb QTL region on chromosome 7 related to salt tolerance at the bud burst stage was identified as the candidate region. Based on RNA sequencing data, the DEGs detected by transcriptome analysis in the candidate region were selected as the candidate genes.

### Candidate Gene Sequencing, Sequence Alignment and Validation of Candidate Genes with Molecular Markers

The corresponding candidate genes in Weiguo and IR36 were cloned using PCR and sequenced. With the genes in the Nipponbare genome as a reference, sequence alignment was carried out using DNAMAN software. The 1 bp Indel difference between IR36 and Weiguo for the candidate gene *Os07g0569700* was used to design the KASP marker (FAM primer: GAAGGTGACCAAGTTCATGCTGGTACCAACTGTTACAAGGAAAAAAAA; HEX primer: GAAGGTCGGAGTCAACGGATTGGTACCAACTGTTACAAGGAAAAAAAAA; Common primer: CCCGGTTAGTATTCAAAGCAAGAT). The marker was further used to genotype 201 individuals, including 199 lines from the F_2:3_ population, which were also used for regional linkage mapping analysis, and the two parents. The method of the KASP assays was the same as that used in regional linkage mapping analysis.

## Supplementary information

**Additional file 1: Figure S1.** Box-plot of phenotypic statistical of SNK in two pools. SNK: Na^+^/K^+^ ratio of shoots.

**Additional file 2: Table S1.** An overview of the QTL-seq results. **Table S2.** SNPs results between two parents and two mixed pools. **Table S3.** Annotated genes within the interval detected by QTL-seq. **Table S4.** Phenotypic variation of parents and F2:3 lines. **Table S5.** Summary of Illumina transcriptome reads mapped to the reference genome. **Table S6.** Statistics of transcriptome sequencing results. **Table S7.** RNA-seq data of the genes in candidate region. **Table S8.** The KASP genotyping results. **Table S9.** ANOVA results of three genotypes. **Table S10.** The Arabidopsis homologous genes of Os07g0569700. **Table S11.** The genotype of 1 bp Indel in 66 diverse rice accessions. **Table S12.** Primers of 25 KASP markers. **Table S13.** The KASP reaction system. **Table S14.** Primers for qRT-PCR in this study.

**Additional file 3: Figure S2.** Volcano plots for expressed genes in the four comparison groups. Volcano plots for all the expressed genes in (A) TWG vs. TIR, (B) WG vs. IR, (C) IR vs. TIR, and (D) WG vs. TWG. X- and Y-axis present the log2 (ratio) for the two samples and -log10 (FDR), respectively. Red (Up regulated) and green (down regulated) dots mean that the genes have significant difference, while the black dots correspond to genes with no significant differences.

**Additional file 4: Figure S3.** Venn diagrams for DEGs in the four comparison groups.

**Additional file 5: Figure S4.** The most significantly-enriched GO terms of DEGs from the four comparison groups. (A) TWG vs. TIR, (B) WG vs. IR, (C) IR vs. TIR, (D) WG vs. TWG.

**Additional file 6: Figure S5.** Analysis of KEGG enrichment for DEGs from the four comparison groups. (A) TWG vs.TIR, (B) WG vs. IR, (C) IR vs. TIR, (D) WG vs. TWG.

**Additional file 7: Figure S6.** The 5 genes expression assay in 5 individuals respectively selected from high, low and intermediate phenotype of RSL in199 F_2:3_ population. Fold change: expression data of salt treatment/expression data of control.

**Additional file 8: Figure S7.** The correlation analysis of female parent genotype, male parent genotype and heterozygous genotype with RSL.

## Data Availability

The BSA-seq clean data were uploaded in the NCBI Sequence Read Archive (NCBI SRA) under the accession number SRR10714357, SRR10714358, SRR10714420, SRR10724346. RNA-seq data were deposited to the SRA database of NCBI (SRP238139).

## Abbreviations

BSA: Bulked segregant analysis
QTL: Quantitative trait locus
RIL: Recombinant inbred line
ED: Euclidean distance
DEGs: Differentially expressed genes
InDel: Insertion deletion
KASP: Kompetitive allele-specific PCR
qRT-PCR: Real time quantitative polymerase chain reaction
SNPs: Single nucleotide polymorphisms
GO: Gene ontology
KEGG: Kyoto Encyclopedia of Genes and Genomes
PVE: Phenotypic variation explanation
S-pool: Sensitive pool
T-pool: Tolerant pool
SL: Shoot length
RN: Root number
RL: Root length
RSL: Relative shoot length
RRN: Relative root number
RRL: Relative root length
SNK: Na^+^/K^+^ ratio of shoots
