# Supplementary figures and images for: Identification of a Major QTL and Candidate Gene Analysis of Salt Tolerance at the Bud Burst Stage in Rice (Oryza sativa L.) Using QTL-Seq and RNA-Seq
Source: Rice (N Y). 2020 Aug 10;13:55. doi: 10.1186/s12284-020-00416-1 (PMC7417472; doi:10.1186/s12284-020-00416-1)

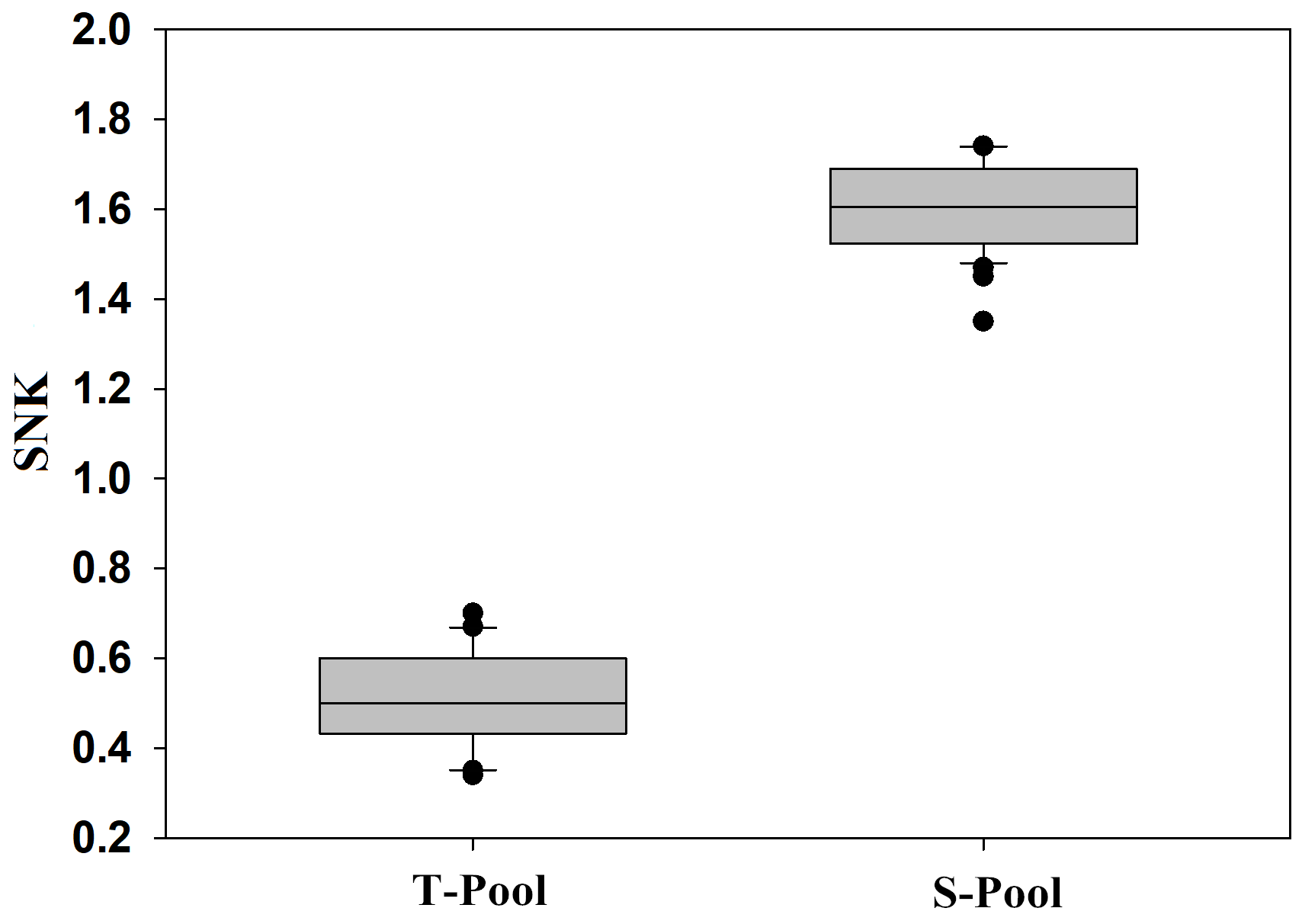

Supplement: Supplementary file 1 — Additional file 1: Figure S1. Box-plot of phenotypic statistical of SNK in two pools. SNK: Na+/K+ ratio of shoots. [file 12284_2020_416_MOESM1_ESM.png]

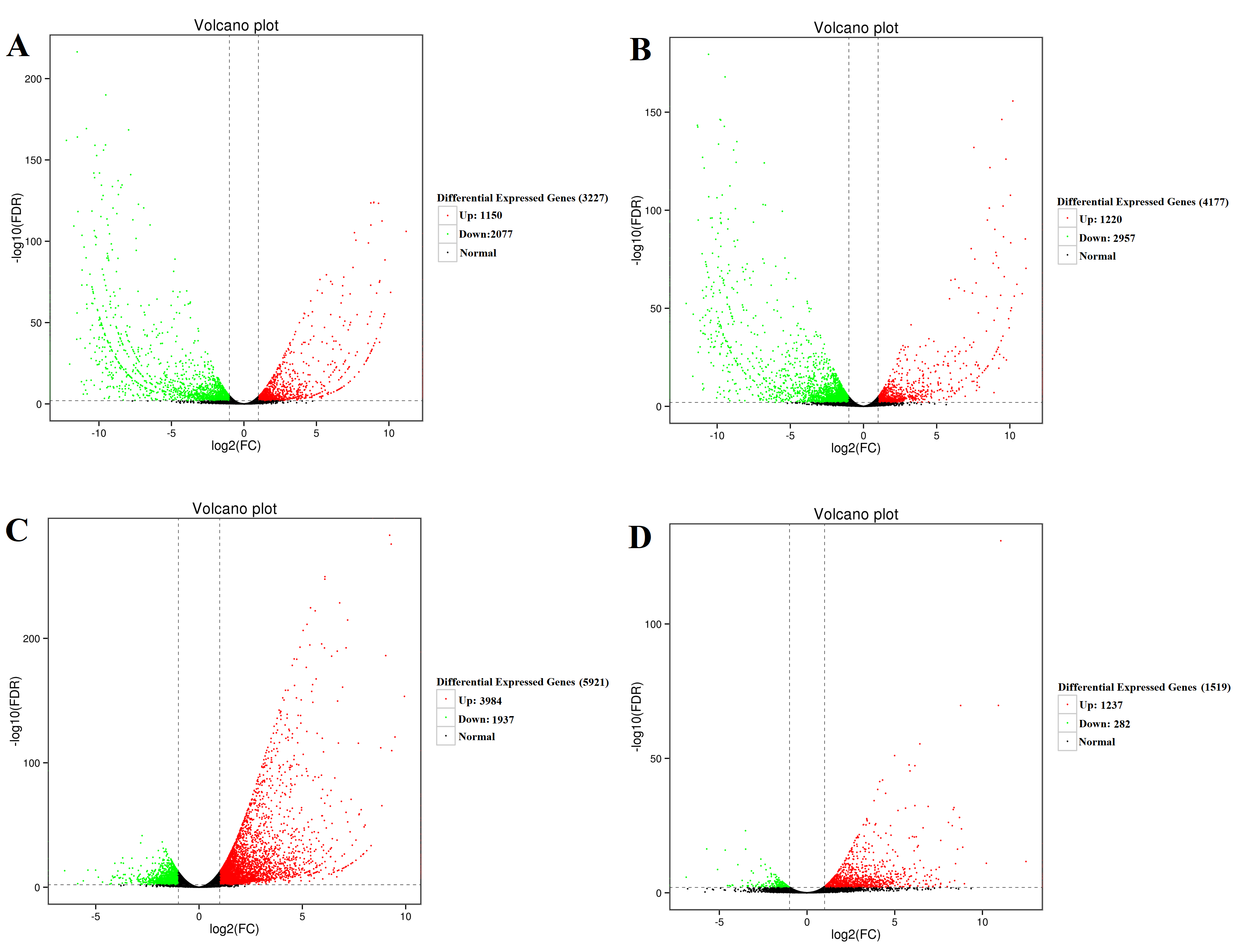

Supplement: Supplementary file 3 — Additional file 3: Figure S2. Volcano plots for expressed genes in the four comparison groups. Volcano plots for all the expressed genes in (A) TWG vs. TIR, (B) WG vs. IR, (C) IR vs. TIR, and (D) WG vs. TWG. X- and Y-axis present the log2 (ratio) for the two samples and -log10 (FDR), respectively. Red (Up regulated) and green (down regulated) dots mean that the genes have significant difference, while the black dots correspond to genes with no significant differences. [file 12284_2020_416_MOESM3_ESM.png]

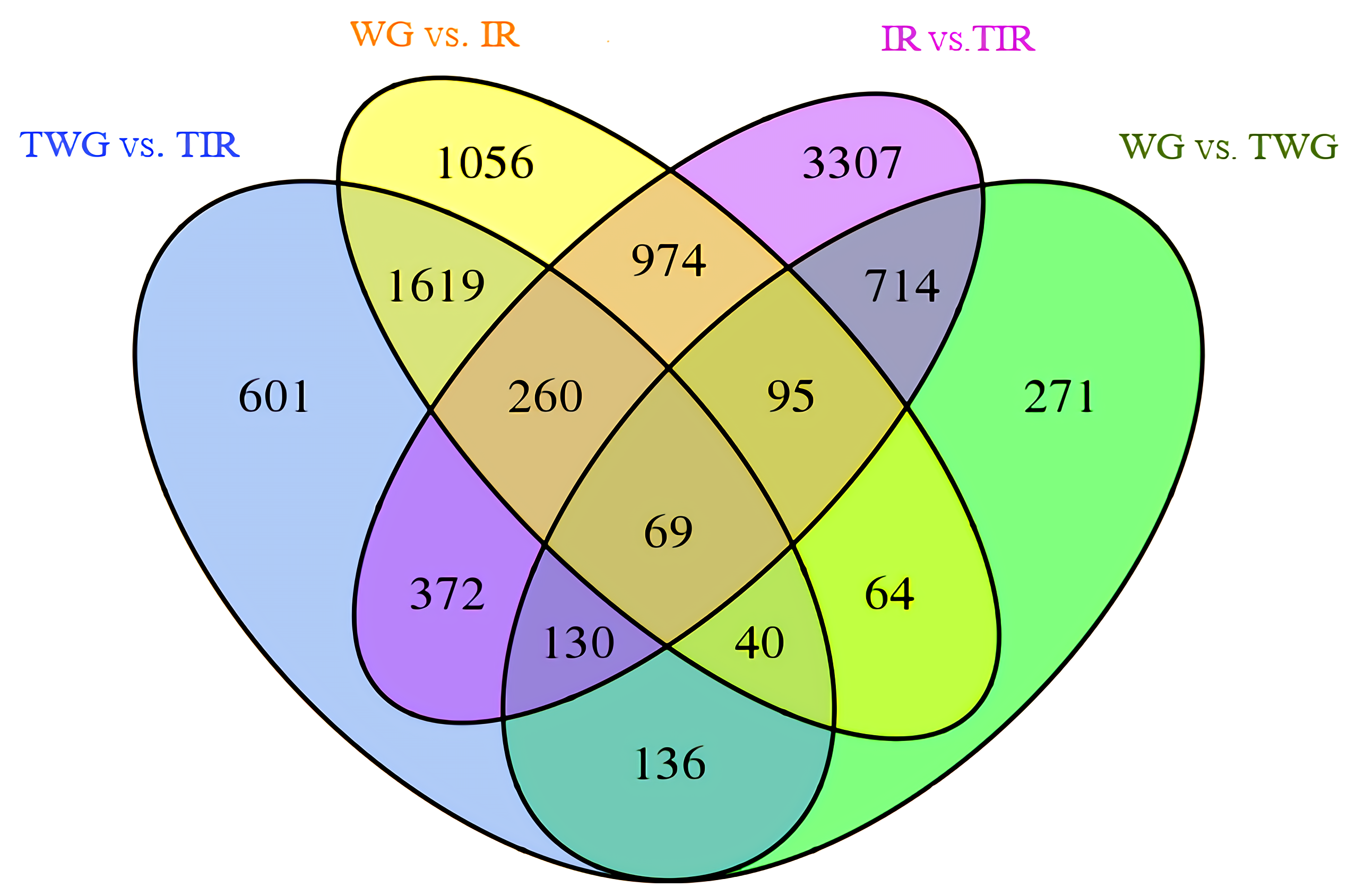

Supplement: Supplementary file 4 — Additional file 4: Figure S3. Venn diagrams for DEGs in the four comparison groups. [file 12284_2020_416_MOESM4_ESM.png]

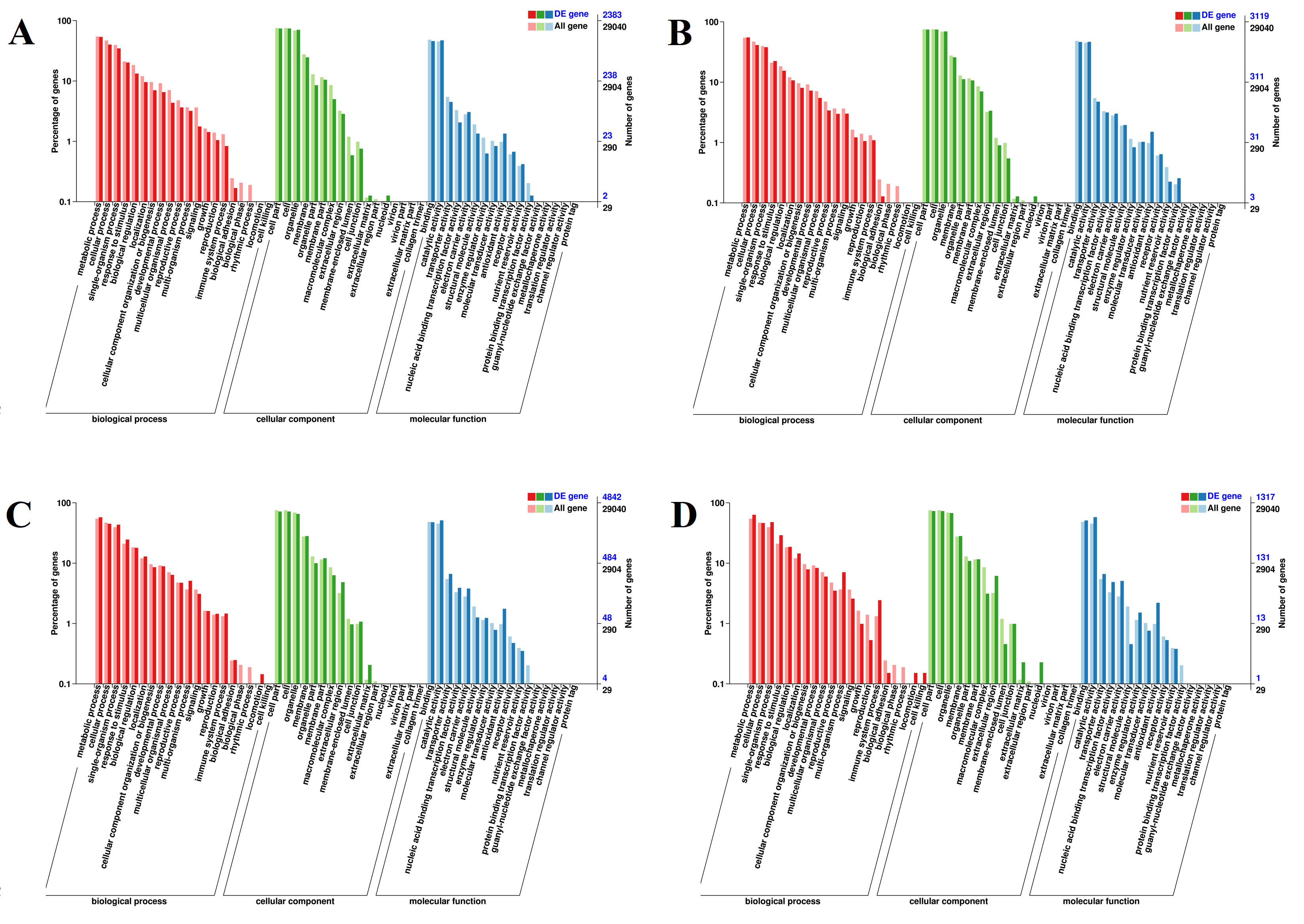

Supplement: Supplementary file 5 — Additional file 5: Figure S4. The most significantly-enriched GO terms of DEGs from the four comparison groups. (A) TWG vs. TIR, (B) WG vs. IR, (C) IR vs. TIR, (D) WG vs. TWG. [file 12284_2020_416_MOESM5_ESM.png]

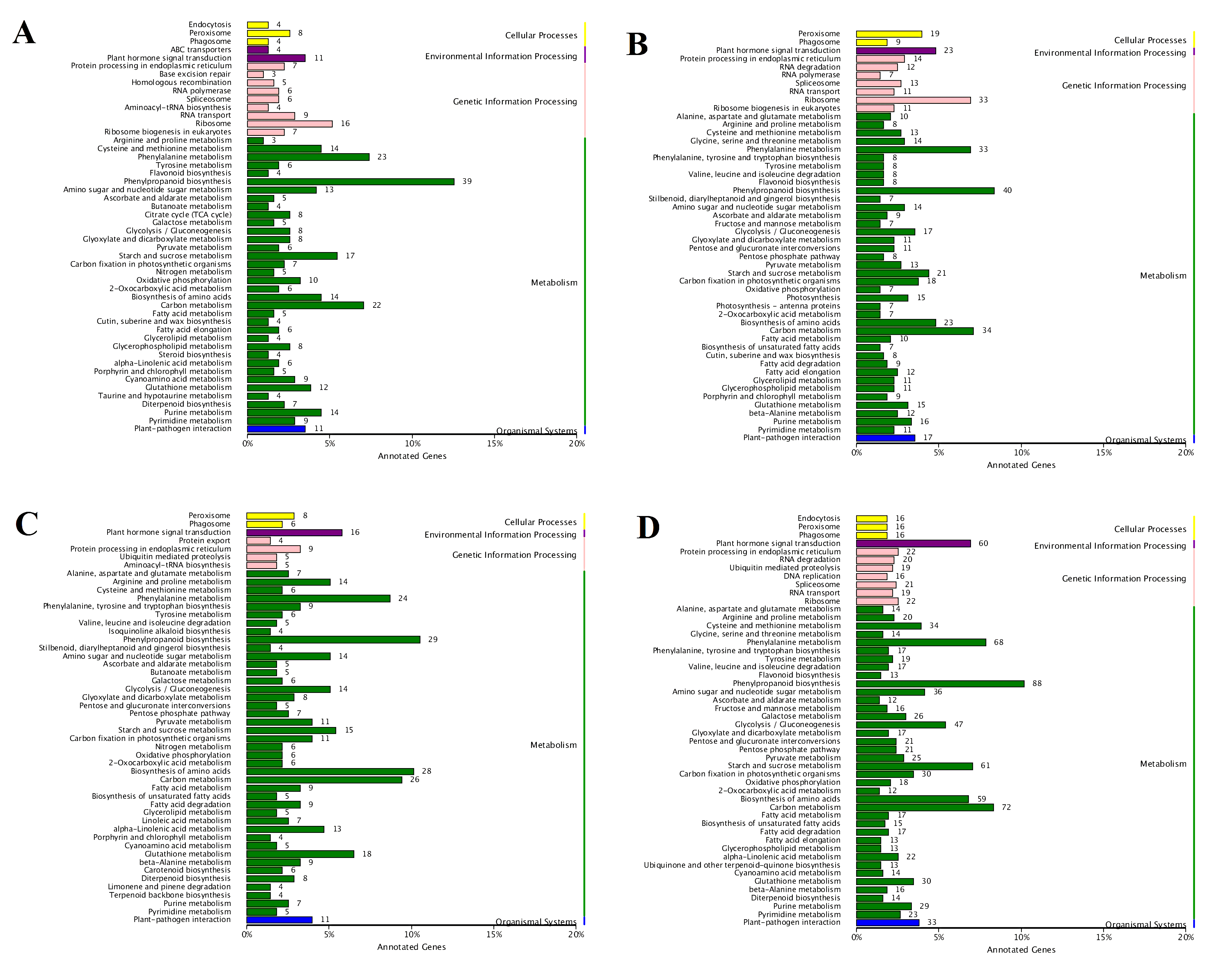

Supplement: Supplementary file 6 — Additional file 6: Figure S5. Analysis of KEGG enrichment for DEGs from the four comparison groups. (A) TWG vs.TIR, (B) WG vs. IR, (C) IR vs. TIR, (D) WG vs. TWG. [file 12284_2020_416_MOESM6_ESM.png]

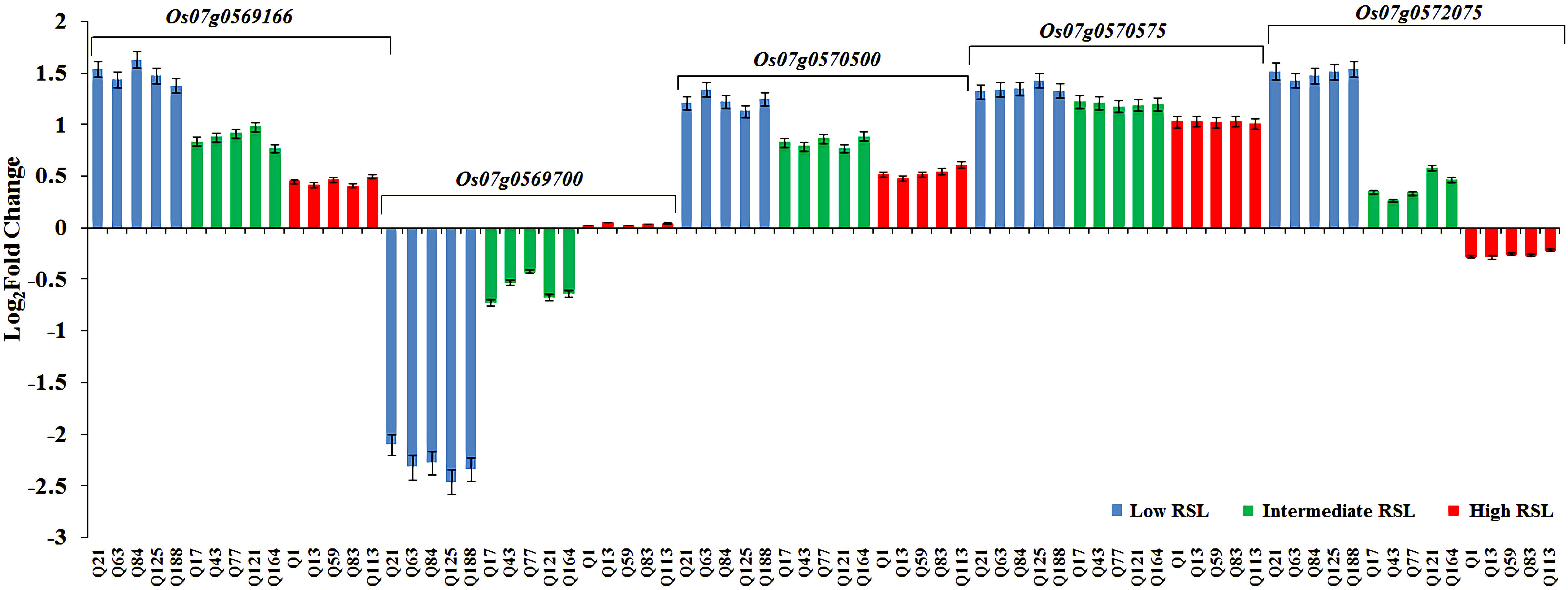

Supplement: Supplementary file 7 — Additional file 7: Figure S6. The 5 genes expression assay in 5 individuals respectively selected from high, low and intermediate phenotype of RSL in199 F2:3 population. Fold change: expression data of salt treatment/expression data of control. [file 12284_2020_416_MOESM7_ESM.png]

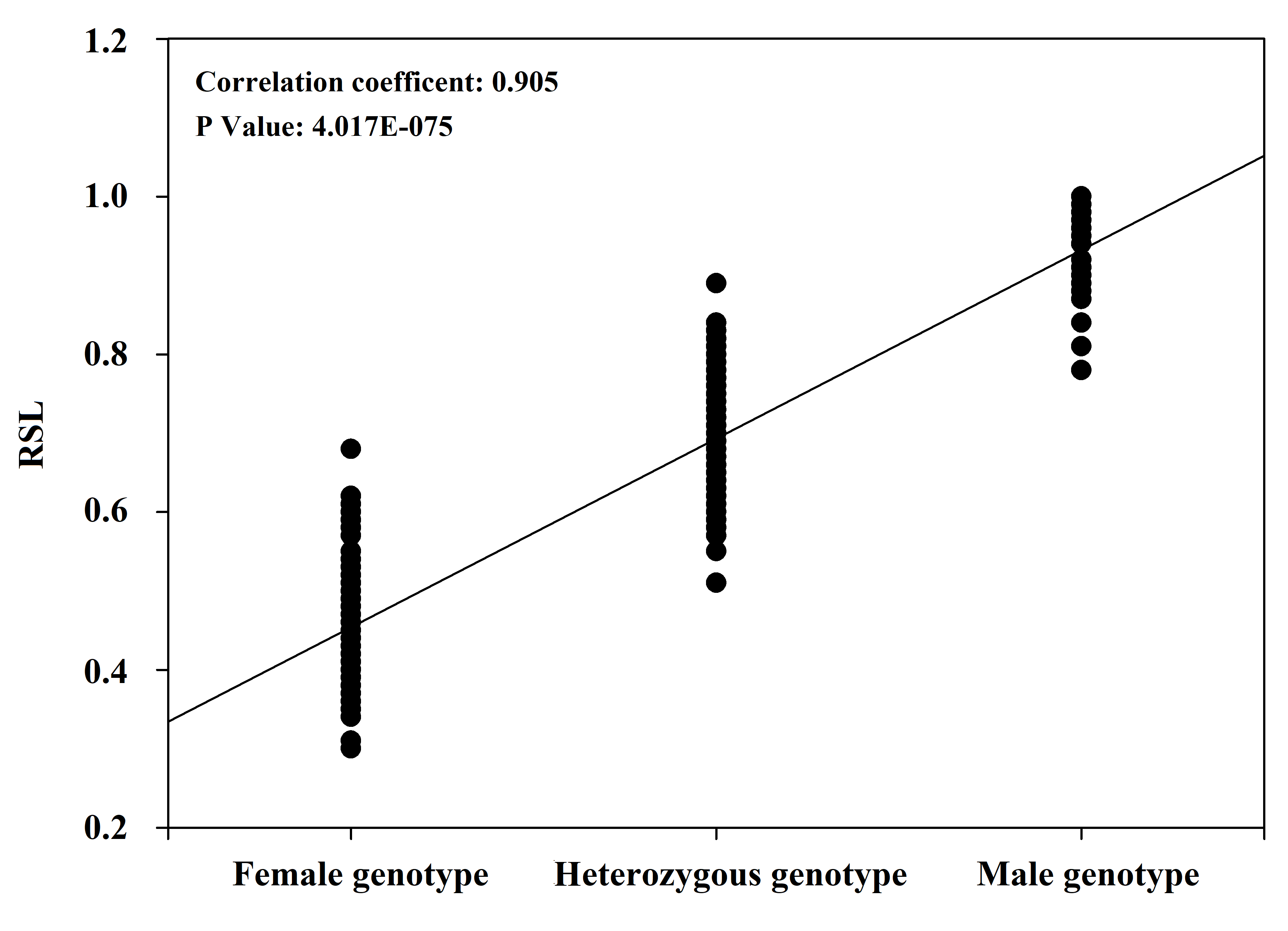

Supplement: Supplementary file 8 — Additional file 8: Figure S7. The correlation analysis of female parent genotype, male parent genotype and heterozygous genotype with RSL. [file 12284_2020_416_MOESM8_ESM.png]
